# Classification, Diagnosis, and Treatment of Coronary Microvascular Dysfunction

**DOI:** 10.3390/jcm11154610

**Published:** 2022-08-08

**Authors:** Vincenzo Sucato, Cristina Madaudo, Alfredo Ruggero Galassi

**Affiliations:** 1 Division of Cardiology, University Hospital Paolo Giaccone, 90127 Palermo, Italy; 2 Department of Health Promotion, Mother and Child Care, Internal Medicine and Medical Specialties (ProMISE), University of Palermo, 90127 Palermo, Italy

Coronary microvascular dysfunction represents a widespread disease which is highly disabling for the patient, who constantly presents angina. Today, it is a challenge for cardiologists who must treat the patient with serious limitations in their daily activities [1]. In patients, especially women, undergoing coronary angiography to evaluate chest pain and stable angina, 20–30% have normal coronary angiograms. This condition is called primary microvascular angina (MVA) in order to distinguish it from forms of MVA in which coronary microvascular dysfunction (CMD) is related to the presence of specific diseases [2]. This pathology affects the pre-arterioles, with a primary involvement of the endothelial function. In recent years, scientific studies have focused on trying to define the disease and identify some pathological settings; currently, MVA can be categorized into a stable form, an unstable form, and a form of acute microvascular dysfunction corresponding to Takotsubo syndrome [3]. There are likely multiple mechanisms responsible for MVA. As for the pathogenesis, there are still many unknown points about MVA; it is not yet clear the importance of some risk factors compared to others, as well as the new alterations of endothelial function [4,5].

Precisely for this reason, today, it is still a topic of both clinical and scientific interest, so much so that the ESC 2019 guidelines on chronic coronary syndrome dedicated a specific section to angina with an absence of significant flow-limiting obstructive coronary artery disease, describing the most frequent variants: vasospastic angina and microvascular angina (Figure 1) [6].

The most recent scientific literature identifies all patients who present symptoms and signs of myocardial ischemia in the absence of coronary obstructive disease (coronary stenosis greater than or equal to 50% of the vessel diameter) in a syndrome defined precisely by the English acronym INOCA (ischemia with non-obstructive coronary arteries) [7].

It is now possible to propose a more updated classification of the various clinical manifestations of ischemic heart disease as shown in Figure 2.

Within the well-known obstructive coronary artery disease, the two new entities of INOCA and MINOCA (myocardial infarction with non-obstructive coronary arteries) appear.

The term INOCA indicates any condition responsible for myocardial ischemia in the absence of hemodynamically significant coronary lesions (stenosis < 50%) after performing angiography.

The angiographic finding of coronary arteries without hemodynamically significant lesions often ends the diagnostic and therapeutic process of these patients who have had symptoms of ischemia and allows them to be identified as healthy subjects. However, recent evidence points out that the prognosis of this patient population is not entirely good, both in terms of the incidence of cardiovascular events and in terms of quality of life. Furthermore, even for clinical and interventional cardiologists, normal coronary ischemia sometimes represents a problem from a pathophysiological point of view but also in terms of the clinical management of the patient.

Up to now, this phenomenon has been studied exclusively through the information that coronary angiography can offer and not considering the vitality of the coronaries in terms of vasoreactivity and microcirculation which, due to obvious technical limitations, cannot be observed with angiography.

Various methods have been proposed for the analysis of the functional state of coronary microcirculation. CMD can only be identified indirectly through measurements of changes in coronary blood flow and coronary vascular resistance in response to particular triggers.

Invasive methods such as thermodilution and intracoronary Doppler are the gold standard. Transthoracic Doppler echocardiography allows measuring the speed of coronary flow and is considered an indirect measure of coronary flow.

Coronary flow reserve (CFR) is measured as the ratio of peak diastolic flow rate during vasodilator infusion (mainly adenosine) to basal flow rate. A ratio < 2.0 is considered diagnostic of coronary microcirculatory dysfunction.

Myocardial scintigraphy is a diagnostic test that is used to evaluate the presence of possible abnormalities in myocardial perfusion. Instead, the evaluation of myocardial perfusion, using cardiac magnetic resonance imaging (RMC), is based on changes in the intensity of the gadolinium myocardial signal.

The TIMI frame count is a quantitative index that allows evaluating how coronary flow is the number of frames necessary to opacify a predetermined distal landmark. The myocardial blush grade indicates the ability of the coronary microcirculation to be opacified by the contrast.

Compared with these methods, the introduction into clinical practice of blood biomarker detection in patients with suspected MVA can facilitate the diagnosis of this pathological condition, offering several advantages, such as a reduction in costs, less risk of physical harm, a greater availability/accessibility, and prognostic value [8].

The data from the American WISE registry (Women’s Ischemic Syndrome Evaluation) indicate, above all, a higher prevalence of INOCA in women [9]. These data show, during follow-up, an intermediate risk of major adverse cardiac events (MACE: death, non-fatal heart attack, non-fatal stroke, hospitalization for heart failure): greater than 2.5% per year in the first 5 years after diagnosis. The rate of re-hospitalizations or repeat diagnostic tests including coronary angiography due to an exacerbation of symptoms has also increased. At 10 years, the risk of cardiovascular death or myocardial infarction rises to 6.7%. The re-hospitalization rate is also higher in women than in men (up to four times higher).

Myocardial infarction with non-obstructive coronary arteries (MINOCA) is defined by clinical evidence of myocardial infarction (MI) with normal or near-normal coronary arteries upon angiography. This condition is present in about 5% to 25% of patients presenting with acute coronary syndromes. MINOCA is a working diagnosis. The current guidelines and consensus recommend identification of the underlying causes of MINOCA in order to optimize treatment, improve prognosis, and promote the prevention of recurrent myocardial infarction. An accurate evaluation of patient history, symptoms, and use of invasive and non-invasive imaging should lead to the identification of epicardial or microvascular causes of MINOCA and differentiation from non-ischemic myocardial injury due to both cardiac (e.g., myocarditis) and non-cardiac disease (e.g., pulmonary embolism) [10].

In conclusion, not only atherosclerotic disease of the epicardial vessels, but also alterations in coronary vasoreactivity and coronary microvascular function, can cause myocardial ischaemia.

The treatment of these patients changes on a case-by-case basis with a tailored treatment strategy. Beta blockers, ACE inhibitors, and statins, in addition to the control of cardiovascular risk factors and lifestyle changes, are indicated for the treatment of microvascular angina. In patients with epicardial or microcirculatory vasomotor disorders, CCBs and long-acting nitrates constitute the treatment of choice.

In recent decades, new nosological entities have emerged such as coronary vasospasm, microvascular spasm, and microvascular angina. The correct definition of these disorders is not yet fully understood in physiopathological terms, and the knowledge and identification of them in daily clinical practice are still not well understood. However, coronary microvascular dysfunction is a disease with a strong impact in a patient’s quality of life, so greater efforts are needed to better understand these pathologies to optimize the diagnostic and therapeutic pathway of these patients [11].

## Figures and Tables

**Figure 1:**
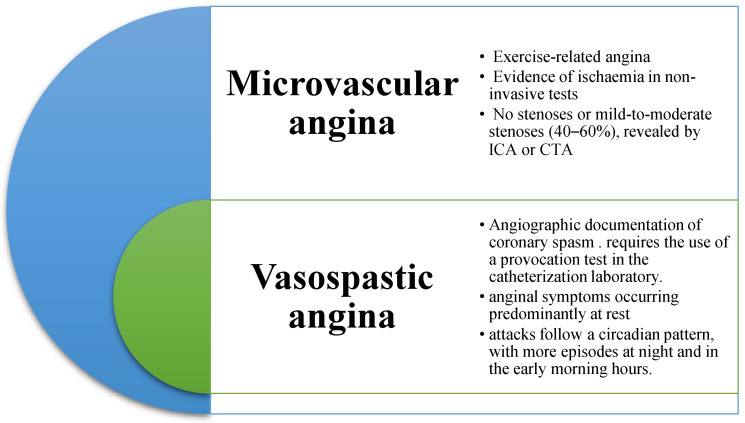
Angina without obstructive disease in the epicardial coronary arteries.

**Figure 2:**
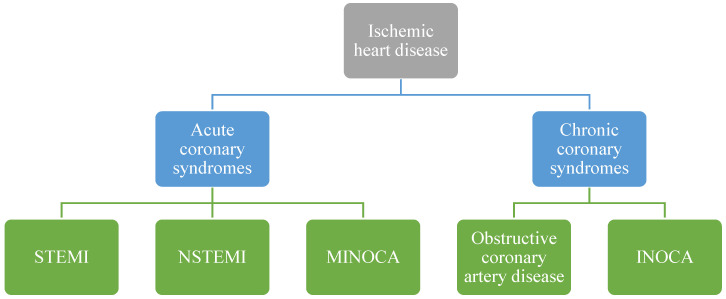
Classification of the various manifestions of ischemic heart disease.
